# The Constituents of *Michelia compressa* var. *formosana* and Their Bioactivities

**DOI:** 10.3390/ijms150610926

**Published:** 2014-06-17

**Authors:** Yu-Yi Chan, Shin-Hun Juang, Guan-Jhong Huang, Yu-Ren Liao, Yu-Fon Chen, Chia-Che Wu, Hui-Ting Chang, Tian-Shung Wu

**Affiliations:** 1 Department of Biotechnology, Southern Taiwan University of Science and Technology, Tainan 71005, Taiwan; 2 Graduate Institute of Pharmaceutical Chemistry, China Medical University, Taichung 40402, Taiwan; E-Mail: paul@mail.cmu.edu.tw; 3 Department of Pharmacy, China Medical University, Taichung 40402, Taiwan; E-Mail: gjhuang@mail.cmu.edu.tw; 4 Department of Chemistry, National Cheng Kung University, Tainan 70101, Taiwan; E-Mail: truthloveroy@yahoo.com.tw; 5 Department of Life Sciences, National Cheng Kung University, Tainan 70101, Taiwan; E-Mail: yufons@gmail.com; 6 School of Forestry and Resource Conservation, National Taiwan University, Taipei 10617, Taiwan; E-Mails: ujin1205@gmail.com (C.-C.W.); chtchang@ntu.edu.tw (H.-T.C.)

**Keywords:** *Michelia formosana*, Magnoliaceae, NO inhibition, macrophage, cytotoxicity

## Abstract

Phytochemical investigation of the heartwood of *Michelia compressa* afforded forty-four compounds, which were identified by comparison of experimental and literature analytical and spectroscopic data. Some compounds were evaluated for their anti-inflammatory and anticancer bioactivities. The result showed that soemerine (**1**) and cyathisterol (**2**) exhibited significant nitric oxide (NO) inhibition, with IC_50_ values of 8.5 ± 0.3 and 9.6 ± 0.5 µg/mL, respectively. In addition, liriodenine (**3**) and oliveroline (**4**) exhibited cytotoxicity to human nasopharyngeal carcinoma (NPC-TW01), non-small cell lung carcinoma (NCI-H226), T cell leukemia (Jurkat), renal carcinoma (A498), lung carcinoma (A549) and fibrosarcoma (HT1080) cell lines with IC_50_ values in the range of 15.7–3.68 μM.

## 1. Introduction

*Michelia* (Magnoliaceae) consists of about 30 species, living in subtropical and tropical Asia, from eastern India through the Indo-Chinese Peninsula to Southern China, Southern Japan and southward to Malay Islands. One species is native to Taiwan [1]. *Michelia* species have been used in the treatment of cancer by indigenous peoples. Such as, *Michelia champaca* has been used in India for the treatment of abdominal tumors as well as *Michelia hypoleuca* and *Michelia officinalis* for carcinomatous sores and leukemia respectively, in China. Experimentally, *Michelia grandiflora*, *Michelia compressa* and *Michelia kobus* have proven anticancer activity in various tumor systems [2]. In addition, the extract of *Michelia champaca* leaves has also exhibited an anti-inflammatory activity in pro-inflammatory conditions [3]. Moreover, the aporphine alkaloid and sesquiterpene lactone constituents of this genus have caused a great interest due to their diverse structures and significant biological activities [2,3,4,5,6].

*Michelia compressa* var. *formosana* is an evergreen tree mainly distributed in Taiwan, Japan, and Ryukyu Islands [1]. In the previous literature, its heartwood exhibited high resistance against rots due to the presence of liriodenine [6]. The chemical investigations of leaves [5,7], barks [8], root barks [2], root wood [4], heartwood [9,10], stems [11], and pericarps [12] have been reported, but the anti-inflammatory and cytotoxic properties of constituents from heartwood are not known.

In our future research, we focus on the identification of anticancer and anti-inflammatory drug leads from natural sources. In our research, the bioactive constituents of the heartwood of *M. compressa* were searched for by assaying the effects on nitric oxide (NO) inhibition in LPS (lipopolysaccharide)-activated mouse peritoneal macrophages and evaluating their cytotoxicity of six human cancer cell lines including human nasopharyngeal carcinoma (NPC-TW01), non-small cell lung carcinoma (NCI-H226), T cell leukemia (Jurkat), renal carcinoma (A498), lung carcinoma (A549) and fibrosarcoma (HT1080) cell lines.

## 2. Results and Discussion

### 2.1. Isolation and Characterization of Compounds

Air-dried and powdered heartwood of *M. compressa* var. *formosana* was soaked with ethanol at room temperature, and the combined extracts were concentrated to give a deep brown syrup. The crude extract was suspended into water and partitioned with CHCl_3_ to afford CHCl_3_ layer and water solubles, respectively. Purification of the CHCl_3_ soluble fraction by column chromatography yielded roemerine (**1**, 13.7 mg) [13], cyathisterol (**2**, 10.8 mg) [14], liriodenine (**3**, 379 mg) [10], oliveroline (**4**, 2.1 mg) [15], ushisurine (**5**, 230 mg) [10], dihydrocostunolide (**6**, 3.6 mg) [16], (−)-*N*-formylanonaline (**7**, 7.9 mg) [15], 6α-hydroxy-β-sitostenone (**8**, 4.7 mg) [15], ferulic acid (**9**, 5.9 mg) [17], β-sitosterone (**10**, 10.3 mg) [18], β-stigmastenone (**11**, 11.5 mg) [18], costunolide (**12**, 1.2 mg) [19], β-sitostanone (**13**, 1.6 mg) [18], dehydroroemerine (**14**, 1.9 mg) [20], ferruginol (**15**, 1.2 mg) [21], sugiol (**16**, 1.3 mg) [22], (^11^*S*)-1-oxoedudesm-4(14)-eno-13, 6α-lactone (**17**, 1.1 mg) [23], dihydroreynosin (**18**, 1.2 mg) [23], romucosine (**19**, 1.6 mg) [24], 11,13-dihydrosantamarin (**20**, 0.8 mg) [25], 2-methylanthraquinone (**21**, 1.5 mg) [26], methyl 22-feruloyloxydocosanoate (**22**, 1.9 mg) [27], annonbraine (**23**, 1.1 mg) [28], ferulic aldehyde (**24**, 0.7 mg) [29], secoroemerine (**25**, 0.6 mg) [30], blumenol A (**26**, 1.3 mg) [31], (−)-*N*-formyldehydroanonaline (**27**, 1.7 mg) [32], 6α,7-dehydroanonaline (**28**, 0.9 mg) [33], *N*-*trans*-feruloyltyramine (**29**, 1.0 mg) [27], *N*-*trans*-feruloylmethoxytyramine (**30**, 1.1 mg) [17], syringaresinol (**31**, 1.7 mg) [34], cepharadione-A (**32**, 0.9 mg) [35], aristolic acid II (**33**, 0.8 mg) [17], *O*-acetyl-ushinsunin (**34**, 0.9 mg) [10], (+)-isolaricirensinol (**35**, 1.6 mg) [34], northalifoline (**36**, 1.5 mg) [35], cassyformin (**37**, 2.0 mg) [36], 3-[2-(4-hydroxy-3-methoxyphenyl)-3-hydroxymethyl-7-methoxy-2,3-dihydro-1-benzofuran-5-yl]propan-1-ol (**38**, 1.7 mg) [37], dehydrodiconiferyl alcohol (**39**, 0.8 mg) [38], divanillyl tetrahydrofuran (**40**, 1.9 mg) [39], methyl vanillate (**41**, 0.7 mg) [24], noroliveroline (**42**, 0.7 mg) [40], 1-acetyl-β-carboline (**43**, 1.6 mg) [41] and *trans*-feruloyloxyhexacosanoic acid (**44**, 2.3 mg) [42], and their structures were identified by comparison of their physical and spectroscopic data with those reported in the literature.

### 2.2. Inhibitory Effects of Isolated Compounds on Nitric Oxide (NO) Production

**Figure 1:**
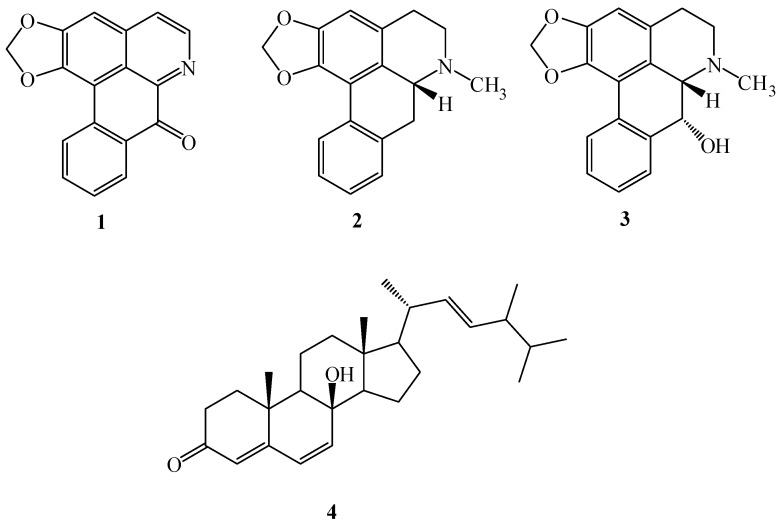
Structure of compounds **1**–**4**.

The isolated compounds (**1**–**3**, **5**, **7**, **9**–**11)** were subjected into the examination of their effects on LPS-induced NO production in murine RAW 264.7 macrophage cell line. RAW 264.7 cells were incubated with various concentrations of these compounds and LPS (100 ng/mL) for 24 h for detection of NO production. NO plays a role as neurotransmitter, vasodilator, and immune regulator in a variety of tissues at physiological concentration. High levels of NO produced by inducible nitric oxide synthase (iNOS) have been defined as a cytotoxic molecule in inflammatory process [43]. After 24 h incubation, the culture medium of non-LPS treated macrophages was used as background levels of nitrite. Nitrite accumulated in the culture medium was estimated by the Griess reaction as an index for NO release from the cells. In the present study, NO production was significantly decreased by the treatment with roemerine (**1**) and cyathisterol (**2**) (Figure 1) in a dose-dependent manner, with IC_50_ values of 8.5 ± 0.3 and 9.6 ± 0.5 µg/mL, respectively (Table 1).

**Table 1:** Inhibitory effects of compounds **1** and **2** from the leaves of *Michelia compressa* var. *formosana* on LPS-induced Nitric Oxide (NO) production in RAW 264.7 cells.

Compound	Dose (100 ng/mL)	Cell Viability (% of Control)	NO Level	IC_50_ (μg/mL)
control	(−)	94.5 ± 2.8	0.7 ± 0.4	–
LPS	(+)	101.7 ± 0.5	19.7 ± 0.2 ^###^	–
1	1.25	97.7 ± 5.1	19.1 ± 1.0	
	2.5	94.7 ± 2.9	18.3 ± 0.5	
	5	89.8 ± 4.8	12.3 ± 0.2 **	8.5 ± 0.3
	10	86.9 ± 2.7	9.1 ± 0.1 ***	
	20	81.3 ± 1.7	6.5 ± 0.3 ***	
2	1.25	85.3 ± 1.9	20.6 ± 0.1	
	2.5	84.3 ± 2.5	20.0 ± 0.5	
	5	82.7 ± 1.7	17.0 ± 0.8 *	9.6 ± 0.5
	10	80.6 ± 5.4	9.4 ± 0.7 ***	
	20	64.5 ± 3.9	(−)	

The data were presented as mean ± S.D; ^###^, compared with sample of control group; *, *p* < 0.05, **, *p* < 0.01, and ***, *p* < 0.001 were compared with LPS-alone group; –, not detectable; (−), no LPS adds to RAW 264.7 cells; (+), LPS adds to RAW 264.7 cells.

### 2.3. Anticancer Bioactivities of Isolated Compounds

To assess the growth inhibitory activity of the isolated compounds **1**–**11** toward cancer cells, six different cell lines from malignant tumors including human nasopharyngeal carcinoma (NPC-TW01), non-small cell lung carcinoma (NCI-H226), T cell leukemia (Jurkat), renal carcinoma (A498), lung carcinoma (A549) and fibrosarcoma (HT1080) cell lines were used. The result showed that liriodenine (**3**) and oliveroline (**4**) (Figure 1) treatment exhibited inhibition of human cancer cell lines with IC_50_ values in the range of 15.7–3.68 μM (Table 2). Moreover, liriodenine (**3**) and oliveroline (**4**) exhibited the powerful inhibitory activity against renal carcinoma (A498) with IC_50_ valuses 4.52 and 3.68 μM, respectively. Our study suggests the heartwood of *M. compressa* var. *formosana* and its isolates could be viewed as potential candidates for the development of anti-cancer drugs.

**Table 2:** Cytotoxicity of compounds **3** and **4**.

	Cell Lines	TW01	H226	Jurkat	A498	A549	HT1080
Compounds		IC_50_ (μM)	IC_50_ (μM)	IC_50_ (μM)	IC_50_ (μM)	IC_50_ (μM)	IC_50_ (μM)
**3**		8.99	14.71	15.7	4.52	8.82	9.75
**4**		5.88	12.28	13.28	3.68	5.50	6.93

## 3. Experimental Section

### 3.1. General Procedures

UV spectra were obtained with a Hitachi UV-3210 spectrophotometer (Hitachi, Tokyo, Japan), and IR spectra were recorded on a Shimadzu Fourier transform infrared spectroscopy (FT-IR) DR-8011 spectrophotometer spectrophotometer (Olympus, Tokyo, Japan). Optical rotations were measured with a JASCO P-2000 digital polarimeter in a 0.5 dm cell. The electrospray ionization mass spectrometry (ESI-MS, Bruker, Bremen, Germany) and high resolution electrospray ionization mass spectroscopy (HRESI-MS, Bruker, Bremen, Germany) were taken on a Bruker Daltonics APEX II 30e spectrometer (Bruker, Bremen, Germany). The ^1^H and ^13^C nuclear magnetic resonance spectroscopy (^1^H and ^13^C NMR) spectra were measured using Bruker Avance-300 (Bruker Biospin Inc., Ettlingen, Germany), AMX-400 (Bruker Biospin Inc., Ettlingen, Germany), and AV-500 spectrometers (Bruker Biospin Inc., Ettlingen, Germany) with tetramethylsilane (TMS) as the internal reference, and chemical shifts are expressed in δ (ppm). Silica gel (70–230 and 230–400 mesh; Merck KGaA, Darmstadt, Germany) and Spherical C18 reversed phase silica gel (RP-18; particle size 20–40 μm; Silicycle, Quebec City, QC, Canada) were used for column chromatography (CC), and silica gel 60 F254 (Merck, Darmstadt, Germany) and RP-18 F254S (Merck) were used for thin layer chromatography (TLC) and preparative TLC, respectively.

### 3.2. Plant Materials

The heartwood of *M. compressa* var. *formosana* (Magnoliaceae) was collected from Taipei Hsien, Taiwan, in June 2012 and verified by Prof. Kuoh, C.-S. A voucher specimen (TSWu-2012006) has been deposited in the Herbarium of National Cheng Kung University, Tainan, Taiwan.

### 3.3. Extraction and Isolation

The heartwood of *M. compressa* var. *formosana* (10 kg) was air-dried and powdered and soaked (24 h) three times with 20 L ethanol at room temperature, and the combined extracts were concentrated under reduced pressure to give deep brown syrup (720 g). The crude extract was suspended into water (1 L) and partitioned five times with 1 L CHCl_3_ to afford CHCl_3_ layer (245 g) and water solubles (462 g), respectively, after removal of the corresponding solvent.

The CHCl_3_ soluble extracts were purified by silica gel column chromatography eluted with CHCl_3_ and MeOH gradients to afford 7 fractions. Fraction 2 was subjected to silica gel column chromatography eluted with *n*-hexane/acetone (25:1) to yield **2**, **5**, **6**, **10**–**21**, and **43**. Purification of fraction 4 by column chromatography with silica gel was eluted by step gradients of *n*-hexane/acetone (15:1 and 9:1) to afford **7** and **22**–**24**. Fraction 5 was subjected to silica gel column chromatography eluted with *n*-hexane/acetone (15:1) to yield **3**, **8**, **25**–**33**, and **44**. Separation of fraction 7 by column chromatography with silica gel eluted by *n*-hexane/acetone (7:1) yielded **1**, **4**, **9**, and **34**–**42**.

### 3.4. Determination of Inhibitory Effects on NO Production

#### 3.4.1. Chemicals

LPS (endotoxin from *Escherichia coli*, serotype 0127:B8), MTT (3-[4,5-dimethylthiazol-2-yl]-2,5-diphenyltetrazolium bromide) and other chemicals were purchased from Sigma Chemical Co. (St. Louis, MO, USA).

#### 3.4.2. Cell Culture

A murine macrophage cell line RAW 264.7 (Bioresources Collection and Research Center, BCRC No. 60001) was purchased from the BCRC of the Food Industry Research and Development Institute (Hsinchu, Taiwan). Cells were cultured in plastic dishes containing Dulbecco’s Modified Eagle Medium (DMEM, Sigma, St. Louis, MO, USA) supplemented with 10% fetal bovine serum (FBS, Sigma) at 37 °C in 5% CO_2_/air and subcultured every 3 days at a dilution of 1:5 using 0.05% trypsin–0.02% EDTA in Ca^2+^- and Mg^2+^-free Dulbecco’s phosphate-buffered saline (DPBS).

#### 3.4.3. Cell Viability Assay

Cells were cultured in 96-well plates at a density of 2 × 10^5^ cells/well in 10% FBS DMEM medium and treated with compounds (**1**–**3**, **5**, **7**, **9**–**11**) in the presence of LPS (100 ng/mL) for 24 h. Cells were washed twice with DPBS and incubated with MTT (100 µL, 0.5 mg/mL) for 2 h to analyze cell viability. The medium was discarded and dimethyl sulfoxide (DMSO) (100 µL) was added. After 0.5 h incubation, absorbance at 570 nm was read using a microplate reader (Molecular Devices, Orleans Drive, Sunnyvale, CA, USA).

#### 3.4.4. Measurement of Nitric Oxide/Nitrite

The presence of nitrite (a metabolite of NO) in the culture medium was analyzed with the Griess assay (Sigma-Aldrich) as described previously [44]. Briefly, cells cultured in 96-well plates were treated with compounds in the presence of LPS (100 ng/mL) at 37 °C for 24 h, and cultured supernatant (100 μL) was mixed with the same volume of Griess reagent (1% sulfanilamide, 0.1% naphthyl ethylenediamine dihydrochloride and 5% phosphoric acid). After incubation at room temperature for 10 min, the absorbance was measured at 540 nm with a Micro-Reader (Molecular Devices, Orleans Drive, Sunnyvale, CA, USA). Serum samples from mice were diluted four times with distilled water and deproteinized by adding 1/20 volume of zinc sulfate (300 g/L) to a final concentration of 15 g/L. After centrifugation at 10,000× *g* for 5 min, 100 μL supernatant was applied to a microtiter plate well, followed by adding 100 μL of Griess reagent. After 10 min of color development, the absorbance was measured at 540 nm. Various concentrations of sodium nitrite were used to perform a standard curve.

#### 3.4.5. Statistical Analysis

Experimental results were presented as the mean ± standard deviation (SD) of three parallel measurements. Statistical evaluation was carried out by one-way analysis of variance (ANOVA followed by Scheffe’s multiple range tests). Statistical significance is expressed as * *p* < 0.05, ** *p* < 0.01 and *** *p* < 0.001.

### 3.5. Determination of the Anticancer Bioactivity

#### 3.5.1. Cell Lines

Human cancer cell lines, including T cell leukemia (Jurkat); non-small cell lung carcinoma (NCI-H226), renal carcinoma (A498), lung carcinoma (A549) and fibrosarcoma (HT1080) were obtained from the American Type Culture Collection (Rockville, MD, USA). A nasopharyngeal carcinoma (NPC-TW01) cell line was purchased from Food Industry Research and Development Institute (Hsinchu, Taiwan). Tumor cells were maintained in proper medium supplemented with 10% fetal bovine serum (FBS) at 37 °C in a humidified atmosphere of 5% CO_2_.

#### 3.5.2. Growth Inhibition Assay

The evaluation of cell growth and survival was carried out according to Hansen *et al.* [45] with modification. Briefly, cells were seeded in a 96-well plates and incubated overnight prior to the addition of the designated compounds at various concentrations for three subculture. Two hours before end of treatment, 15 μL of MTT solution (5 mg/mL) was added to each well, and the cells were incubated at 37 °C for 4 h. Subsequently 75 μL lysis buffer (20% SDS–50% *N*,*N*-dimethyl formamide) was added to each well, and the culture plate was incubated at 37 °C overnight to dissolve the dark blue crystals. The conversion to formazan by metabolically viable cells was measured by absorbance at 570 nm. The percentage of conversion by control cells was used to evaluate the effect of the compounds on cell growth and to determine the IC_50_ values.

## 4. Conclusions

In summary, forty-four compounds were characterized from the heartwood of *Michelia compressa* var. *formosana*. Furthermore, the inhibitory activity on LPS-induced NO production in RAW 264.7 cells and the cytotoxicity on six cancer cells were analyzed. These results provide a potential explanation for the usage of the heartwood of *M. compressa* as the herbal medicine in the treatment of cancer diseases, and they may be potentially useful in developing new anticancer agents.
