# Correction: Chan, Y.-Y., *et al.* The Constituents of *Michelia compressa* var. *formosana* and Their Bioactivities. *Int. J. Mol. Sci.* 2014, *15*, 10926–10935

**DOI:** 10.3390/ijms17050734

**Published:** 2016-05-16

**Authors:** Yu-Yi Chan, Shin-Hun Juang, Guan-Jhong Huang, Yu-Ren Liao, Yu-Fon Chen, Chia-Che Wu, Hui-Ting Chang, Tian-Shung Wu

**Affiliations:** 1 Department of Biotechnology, Southern Taiwan University of Science and Technology, Tainan 71005, Taiwan; 2 Graduate Institute of Pharmaceutical Chemistry, China Medical University, Taichung 40402, Taiwan; paul@mail.cmu.edu.tw; 3 Department of Pharmacy, China Medical University, Taichung 40402, Taiwan; gjhuang@mail.cmu.edu.tw; 4 Department of Chemistry, National Cheng Kung University, Tainan 70101, Taiwan; truthloveroy@yahoo.com.tw; 5 Department of Life Sciences, National Cheng Kung University, Tainan 70101, Taiwan; yufons@gmail.com; 6 School of Forestry and Resource Conservation, National Taiwan University, Taipei 10617, Taiwan; ujin1205@gmail.com (C.-C.W.); chtchang@ntu.edu.tw (H.-T.C.)

The authors wish to make two changes to their published paper [1]. The word “soemerine (**1**)” in the abstract, line 4, needs to be changed to “roemerine (**1**)”. The authors also wish to change the content of Figure 1 as shown below. The authors apologize for any inconvenience these changes may cause.

The changes do not affect the scientific results. The manuscript will be updated and the original will remain online on the article webpage.

## Figures and Tables

**Figure 1:**
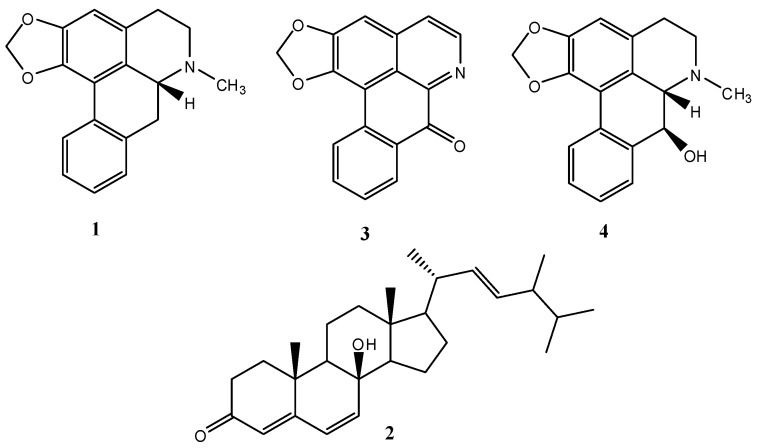
Structure of compounds **1**–**4**.
